# Selective Microfluidic Capture and Detection of Prostate Cancer Cells from Urine without Digital Rectal Examination

**DOI:** 10.3390/cancers13215544

**Published:** 2021-11-04

**Authors:** Kit Man Chan, Jonathan M. Gleadle, Philip A. Gregory, Caroline A. Phillips, Hanieh Safizadeh Shirazi, Amelia Whiteley, Jordan Li, Krasimir Vasilev, Melanie MacGregor

**Affiliations:** 1 Future Industries Institute, UniSA STEM, University of South Australia, Adelaide, SA 5095, Australia; kit_man.chan@mymail.unisa.edu.au (K.M.C.); hanieh.safizadehshirazi@unisa.edu.au (H.S.S.); Krasimir.Vasilev@unisa.edu.au (K.V.); 2 Department of Renal Medicine, Flinders Medical Centre, Bedford Park, SA 5042, Australia; jonathan.gleadle@flinders.edu.au (J.M.G.); jordan.li@sa.gov.au (J.L.); 3 College of Medicine and Public Health, Flinders University, Bedford Park, SA 5042, Australia; 4 Centre for Cancer Biology, University of South Australia and SA Pathology, Adelaide, SA 5000, Australia; Philip.Gregory@unisa.edu.au (P.A.G.); Caroline.Phillips@unisa.edu.au (C.A.P.); 5 Faculty of Health and Medical Sciences, The University of Adelaide, Adelaide, SA 5000, Australia; 6 Ecole Nationale Superieure de Chimie, Bordeaux INP, 33607 Pessac, France; ameliawhiteley97@gmail.com

**Keywords:** urine, prostate cancer, cancer detection, microfluidic, nanotechnologies, biosensors, PSMA, hexaminolevulinic acid, photodynamic diagnosis

## Abstract

**Simple Summary:**

Prostate cancer is the second most common cancer and the fifth leading cause of cancer death in men worldwide. The current diagnosis methods for prostate cancer are invasive and costly. In particular, digital rectal examination (DRE) or prostate massage adds considerable discomfort to patients, reduces compliance to cancer screening schedules, and raises the cost of the diagnostic procedure. New technologies are urgently needed for the effective and yet noninvasive detection of these conditions. This manuscript describes streamlined biotechnology for the noninvasive detection of prostate cancer from malignant cells shed in urine. For the first time, a whole-cell immunocapture approach combined with photodynamic diagnostic principles is used in a device to detect whole cancer cells from unprocessed patient urine samples collected without prior DRE.

**Abstract:**

Urine-based biomarkers have shown suitable diagnostic potential for prostate cancer (PCa) detection. Yet, until now, prostatic massage remains required prior to urine sampling. Here, we test a potential diagnostic approach using voided urine collected without prior digital rectal examination (DRE). In this study, we evaluated the diagnostic performance of a microfluidic-based platform that combines the principle of photodynamic diagnostic with immunocapture for the detection of PCa cells. The functionality and sensitivity of this platform were validated using both cultured cells and PCa patient urine samples. Quantitative reverse-transcriptase polymerase chain reaction (qRT-PCR) demonstrated this platform had a detection limit of fewer than 10 cells per 60 µL and successfully validated the presence of a PCa biomarker in the urine of cancer patients without prior DRE. This biosensing platform exhibits a sensitivity of 72.4% and a specificity of 71.4%, in suitable agreement with qRT-PCR data. The results of this study constitute a stepping stone in the future development of noninvasive prostate cancer diagnostic technologies that do not require DRE.

## 1. Introduction

Prostate cancer (PCa) is the second most commonly diagnosed cancer and the fifth leading cause of cancer death in men worldwide [1]. To date, prostate cancer screening includes digital rectal examination (DRE) and a blood test for prostate-specific antigen (PSA). However, the results from these screening tests do not provide a definite diagnostic but are used to decide whether or not a tissue biopsy examination is warranted [2,3]. Typically, a PSA level above 4 ng mL^−1^ (standard cut-off value) has been accepted as the threshold to recommend a biopsy. Additionally, the IMPACT (Identification of Men with a genetic predisposition to ProstAte Cancer) study proposed annual PSA screening in the population with germline breast cancer 1/2 early-onset (BRCA1/2) and prostate biopsy if PSA level above 3 ng mL^−1^ [4]. However, PSA testing has poor specificity in the range 4–10 ng mL^−1^, which leads to many unnecessary biopsies being conducted [5]. Transrectal ultrasound (TRUS)-guided biopsy is the standard of care for cancer diagnosis. Specifically, international guidelines recommend a core biopsy of 10 to 12 systematic transrectal or transperineal zone biopsies for initial diagnosis [6]. The guidelines also suggest conducting repeat biopsies for men at increased risk of PCa and those with ongoing suspicion of cancer even after previously negative biopsy results [7]. Yet, TRUS is associated with significant pain, discomfort, and risk of hospitalization, for complications ranging from hematuria to bacterial infections [8]. Improved understanding of the genetic and epigenetic predictors of prostate cancer outcomes [9] and strategies to reduce the number of unnecessary biopsies are needed to improve patient wellbeing and reduce costs to the healthcare systems [10].

In this context, urinary liquid biopsy is an attractive and promising alternative for PCa detection. There is also an emerging role in the use of serum biomarkers to detect PCa, including androgen receptor (AR) variants, bone metabolism, neuroendocrine and metabolite biomarkers [11]. Urine readily contains several types of PCa biomarkers, including exosomes, microRNAs [12], DNAs [13], and RNA [14]. Previous works have shown that RNA transcribed from prostate-specific genes and/or prostate cancer genes could be detected in urine or urine sediment [15]. Using the gene expression level of various urinary biomarkers for noninvasive diagnosis of PCa has been trialed in small patient cohorts; however, accuracy was limited [16]. Some studies have investigated the potential of whole PCa cell capture as a way to detect PCa-specific biomarkers from urine [17,18,19]. In most studies investigating the diagnosis potential of urine-based biomarkers, DRE or prostatic massage was conducted prior to urine sampling in order to obtain enough material for PCa detection. Hendriks et al., for instance, reported that the mRNA levels of PCa-related biomarkers (PCA3, KLK3, and ERG) were significantly higher in post-DRE urine compared with the pre-DRE urine [20]. In all cases, urine samples were again collected after DRE, which adds considerably to the cost and reduced acceptability of the test. What remains unclear is whether the amount of PCa cells naturally shed in the urine without prior DRE could in itself be an indicator of advanced PCa.

We previously reported on the development of a microfluidic device capable of capturing PCa cancer cells spiked in healthy urine [21]. In these original works, the PCa cells were specifically captured from cell populations comprising both cancer and healthy cells spiked at a 1:1 ratio. However, without prostatic massage/DRE, the ratio of prostate cancer cells to healthy cells found in patient urine could realistically be much lower. In order to understand the potential cell capture limitations of the developed sensor, in this work, the microfluidic platform was tested with increasingly challenging ratios of cancer vs. normal cells. We then tested patient urine sediments collected without prior DRE for genes associated with prostatic cells. Our findings indicate that the microfluidic immunocapture platform is capable of detecting PCa cells in patient urine samples without the requirement for DRE.

## 2. Materials and Methods

### 2.1. Cell Culture

Human normal prostate epithelium PNT2, prostate carcinoma LNCaP clone FGC and 22Rv1 cells were cultured following CellBank Australia recommendation as detailed in Supplementary Materials and methods.

### 2.2. Preparation of Microfluidic Device

The three plasma deposited polyoxazoline (PPOx)-coated microchannels of each polymethyl methacrylate (PMMA) chip were set as test, negative control, and positive control, respectively. The positive control channel was used as is, with the biocompatible PPOx surface allowing the non-specific attachment of all cell types [22]. The test channel was biofunctionalized with 60 µL of anti-PSMA antibodies (10 µg mL^−1^). Then, 1 mg mL^−1^ skim milk solution, acting as a block solution, was added to both the test and the negative control channels for 45 min. The slides were rinsed with phosphate-buffered saline (PBS) three times, and fresh PBS was added afterward [21]. Following this procedure, the PPOx coating present in the negative control channel is blocked by the skim milk proteins, which prevents the non-specific binding of any cell types. In the test channel, the skim milk blocking step is used to limit non-specific cell binding from happening in between the surface-bound PSMA antibodies.

### 2.3. Cell Capture Efficiency on the Microfluidic Platform

Normal prostate epithelial PNT2 cells and prostate cancer LNCaP cells suspension (2 × 10^5^ cells mL^−1^) were treated with hexaminolevulinate hydrochloride (HAL) (50 µM) and nuclear red (0.5 µM) and used to optimize the specific immunocapture capability of the microfluidic device. The PNT2 and LNCaP cells were mixed together and adjusted with varying ratios (1:1, 1:10, and 1:50) before adding 60 µL to each microchannel for capture and imaging, as detailed in Supplementary Materials and methods.

### 2.4. Patient Sample

The cohort consisted of 50 men enrolled at Flinders Medical Centre and Noarlunga Hospital (Adelaide, SA, Australia) between July 2019 and August 2020 with suspicion of prostate cancer. Histopathology tests diagnosed 29 of the patient with PCa, while 21 had benign prostatic hyperplasia. This study was approved by the Southern Adelaide Clinical Human Research Ethics Committee (HREC/19/SAC/33). Written informed consent was obtained from each patient. Urine samples were collected without previous prostate massage. Clinical pathologic and urinalysis test results are provided in Supplementary Materials and methods. For the clinical trial, the patient urine samples collected without prior DRE were kept at 4 °C and processed within 6 h of collection. A total of 2.5 mL of urine was added to 2.5 mL of PBS with 50 µM HAL. The urine was settled in the dark for 1 h at room temperature. After 1 h incubation, Nuclear red was added to 400 µL of urine sediment to a concentration of 0.5 µM. Each of the microchannels was loaded with 60 µL of urine sample and imaged under a specially designed fluorescence microscope in triplicates. This microscope was equipped with appropriate LED sources, custom filters for PpIX fluorescence measurement, and a field of view larger than a conventional microscope [23,24]. As a result, the whole channel area (68.3 mm^2^) could be imaged in just 150 s, and the resulting image files were reasonable enough in size (600 MB maximum) to be stored for ethics purposes.

In all experiments, the microfluidic device was first imaged immediately after loading the cells into the channels. After 45 min, all microchannels were rinsed off with 1 mL of fresh PBS, and the surfaces were imaged a second time. Fluorescent images in all microchannels were recorded and analyzed by Image-Pro Premier software (Media Cybernetics Inc., Rockville, MD, USA). The evaluation criteria and the test accuracy were measured as previously described [25].

### 2.5. RNA Extraction from Cultured Cells Spiked in Experiment and Patient Urine Samples

For calibration and to determine the limit of detection for low prostate cancer cell numbers in patient urine, cultured cells (concentration range between 2 and 90,000 cells) were spiked into 1 mL of filtered urine from a non-cancer patient urine sample. For actual testing of patient urine samples, different volumes (1, 3, 5 mL) of urine were collected. In both cases, the fluid samples were centrifuged at 1200 rpm for 5 min. The supernatant was discarded, and the cell pellets were resuspended before RNA extraction with the RNeasy Plus Micro Kit (Qiagen, Chadstone Centre, VIC, Australia) according to the manufacturer’s protocol.

PCR was also conducted on both cultured cells and cells present in the patient urine samples following capture in the microchannels. A total of 75 µL of RLT buffer was added to each of the microchannels to lyse the captured cells out. The lysate was transferred to a microcentrifuge tube, and the RNA extraction was then performed with the same kit.

### 2.6. cDNA Synthesis, Preamplification, and qRT-PCR

qRT-PCR was performed to confirm the presence of prostatic cells in the tested clinical samples and lysate of urinary cells captured in the microfluidic device. cDNA was synthesized from the extracted RNA in a reverse transcription reaction using the QuantiTect Reverse Transcription Kit (Qiagen, VIC, Australia) following the manufacturer’s instructions and kept at −20 °C until use. The amount of cDNA was enriched using the TaqMan PreAmp Master Mix Kit and a pool of targeted TaqMan Gene Expression Assays (Thermo Fisher Scientific, Scoresby, VIC, Australia). The test was conducted with androgen receptor (AR), kallikrein-related peptidase 3 (KLK3), prostate cancer-associated 3 (PCA3), prostate-specific membrane antigen (PSMA), and glyceraldehyde-3-phosphate dehydrogenase (GAPDH) probes. Each sample was performed in triplicate. The conditions of preamplification and qRT-PCR are provided in Supplementary Materials and methods.

### 2.7. Development of qRT-PCR Method for Cell Number-Based Unit

Trypsinized prostate cancer LNCaP cells were serial diluted to 2 × 10^5^ cells mL^−1^ with PBS. The cell solution was dispensed into 1 mL of filtered (0.22 µm) Benign prostatic hyperplasia (BPH) patient urine sample with final cell number ranging from 9 × 10^4^ cells mL^−1^ to 2 × 10^0^ cells mL^−1^. The serially diluted cell solutions were used to build a calibration curve. RNA extraction, cDNA synthesis, preamplification, and qRT-PCR were conducted as described above. Cycle threshold values were measured to obtain a spiked-in calibration curve for each tested gene. The calibration curves were used to evaluate the “equivalent” cell number per mL of patient urine samples (Figure S1).

### 2.8. Statistical Analysis

Statistical analyses were performed using Minitab (version 18, Coventry CV3 2TE, UK), OriginPro (version 9.6 Northampton, MA, USA), and MedCalc (19.2.6, MedCalc Software Ltd., Ostend, Belgium) programs. Welch’s two-tailed t-test was applied to compare the unequal variances, and the Mann–Whitney U test was used as a nonparametric alternate analysis to compare between the BPH and PCa groups. All analyses with *p* < 0.05 were considered statistically significant. Normalized mRNA expression levels (CT target—CT housekeeping) and also presence/absence status were analyzed. In qRT-PCR experiments, statistical evaluations were performed with 2^−ΔCT^ values [26,27]. The area under the ROC curve (AUC) with standard error and 95% confidence interval (CI), the sensitivity, specificity, positive and negative predictive values (PPV and NPV) of the panel were assessed by the receiver operation curve (ROC) analysis.

## 3. Results

### 3.1. Specific Capture of Cultured PCa Cells in the Microfluidic Device

**Figure 1:**
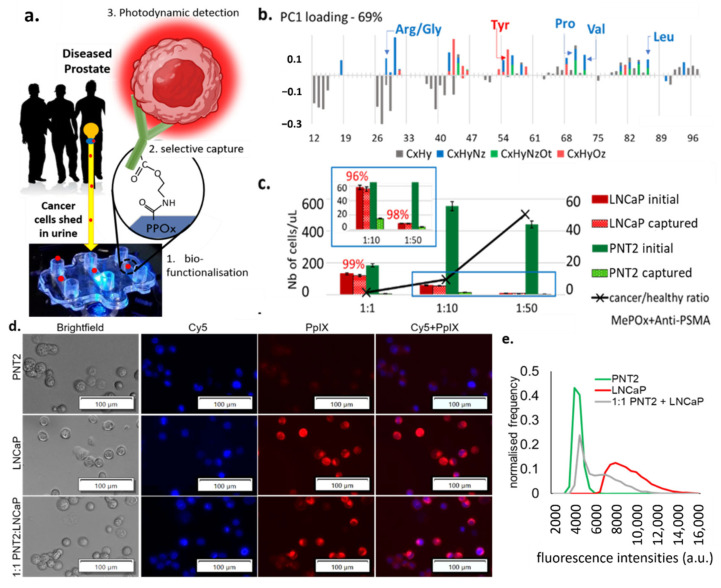
(**a**) Schematic representation of the cell capture device showing the 3 essential steps to its working principle: 1. the microchannel biofunctionalization; 2. the selective cell capture, and 3. the HAL-induced photodynamic detection. (**b**) ToF SIMS principal component analysis of the greatest variance between the pristine PPOx coating and the PPOx following the biofunctionalization with anti-PSMA antibody. Arg/Gly, arginine/glycine; Tyr, tyrosine; Pro, proline; Val, valine; Leu, leucine; CxHy, compounds contain C, H; CxHyNz, compounds contains C, H, N; CxHyNzOt, compounds contains C, H, N, O; CxHyOz, compounds contains C, H, O. (**c**) Numbers of prostate cells captured in the biofunctionalized microchannels for cancer to normal prostate cell ratio 1:1, 1:10 and 1:50, showing the selective cell capture % in red. MePOx, methyl polyoxazoline. (**d**) microscopic images showing the PpIX fluorescence in prostate cancer LNCaP and in mix cell suspension after being treated with HAL compared to normal prostate PNT2 cells. Scale bars represent 100 µm, magnification 10×. Cy5, cyanine-5. (**e**) corresponding fluorescence intensity histograms, showing distinct peaks of maximum fluorescence for healthy and PCa cells.

The microfluidic device was optimized as previously reported [21]. Briefly, plastic molded fluidic chambers (Motherson Innovation Australia, Adelaide, SA, Australia) were coated with a 50 nm thick layer of plasma deposited polyoxazoline (PPOx). This biocompatible polymeric film contains oxazoline rings [28,29] and has been shown to facilitate the irreversible binding of antibodies via reaction with the carboxylic acid groups present on the biomolecules, as shown schematically in Figure 1a [30,31]. Here the PPOx films were functionalized with anti-PSMA antibodies in order to selectively bind PCa cells from urine. PSMA is a membrane-bound glycoprotein (encoded by the FLH1 gene) known to be highly enriched in PCa [32]. Accordingly, it is widely used in PCa imaging, with several studies investigating its potential utility as a diagnostic marker and therapeutic for PCa [33,34,35]. The biofunctionalization of the PPOx coatings with anti-PSMA was assessed via TOF SIMS, Figure 1b. time-of-flight secondary ion mass spectrometry (ToF SIMS) detects low molecular weight fragments present from the topmost 2 nm of the substrates and can therefore be used to distinguish pristine from biofunctionalized PPOx films.

In this analysis, all organic (C, N, O, H) fragments with nominal weight below 100 m z^−1^ were considered, which includes those specific to amino acids. Analysis of the greatest variance (principal component analysis) across the data set was used to identify the molecular fragment that distinguished pristine PPOx films from biofunctionalized ones [22]. The PCA denoted the presence of fragments corresponding to various amino acids on the biofunctionalized PPOx surface that were not present on the pristine one. Specifically, the presence of fragments associated with leucine (C_5_H_10_N^+^), valine (C_4_H_10_N^+^), and proline (C_4_H_8_N^+^) confirmed the successful biofunctionalization with anti-PSMA. These test microchannels were subsequently blocked with skim milk proteins to limit non-specific binding. In preliminary works, we demonstrated the ability of the biofunctionalized chip to bind prostate cancer cells with up to 97% sensitivity [21]. Here we tested the selectivity of the chip using mixed cell suspensions with an increasingly challenging ratio of cancer (LNCaP) to normal (PNT2) prostate cells. The results of the selective cell capture experiments are shown in Figure 1c. For all cell ratios investigated, in excess of 95% of the LNCaP cell were selectively captured. In contrast, only between 1% and 6% of the normal PNT2 cells remain bound to the substrate post rinse. This is in suitable agreement with previous work, which showed, via western blots, that LNCaP expresses PSMA while PNT2 does not [21]. While this result demonstrates the capacity of the device to enrich [36] the population of cancer cells by >90%, it also highlights the need for subsequent cancer-specific detection, as the residual amount of normal cells’ non-specific binding can not be completely avoided.

Following the prostate cells immunocapture, their presence in the microchannel is detected via fluorescence microscopy based on their HAL-induced protoporphyrin IX (PpIX) fluorescence intensity. HAL is the lipophilic hexylester of 5-ALA, a precursor of PpIX in the heme biosynthetic pathway [37]. The exogenous administration of 5-ALA increases the endogenous accumulation of PpIX preferentially in tumor cells of various origins, a property that has been exploited for cancer photodynamic diagnostic (PDD) and therapies (PDT) [21,38,39,40,41]. More recently, HAL-induced PpIX fluorescence has been shown to assist in the ex vivo detection of urogenital malignancies in urine sediment [42,43].

Here, the intensity of HAL-induced PpIX fluorescence was measured in suspensions of LNCaP, PNT2, and 1:1 mix of these cells. Zoomed-in images of captured LNCaP and PNT2 cells (magnification 10×) showing details of the fluorescent features are provided in Figure 1d and zoomed out the field of view in Figure S2. Using the cell counting program built in the Image-Pro Premier software, the mean fluorescence intensity of each object was recorded after background exclusion. Objects with comparable fluorescence intensity were grouped to construct a normalized histogram, representing the average statistical distribution of the cells’ fluorescence intensity in each sample. LNCaP and normal prostate PNT2 cells were found to have distinctly resolved PpIX fluorescence intensity peaks, with LNCaP displaying higher fluorescence intensity (Figure 1e). The peak of the intensity profile in PNT2 closely matches the first intensity peak of the mixed cell suspension group, with the tailing peak of the mixed cell suspension group shifting toward higher fluorescence intensities. Collectively, these data indicate that LNCaP cancer and PNT2 normal prostate cells exhibit distinct fluorescence intensities upon HAL administration.

### 3.2. PCR Assay Sensitivity with Low Numbers of Cultured Cells

**Figure 2:**
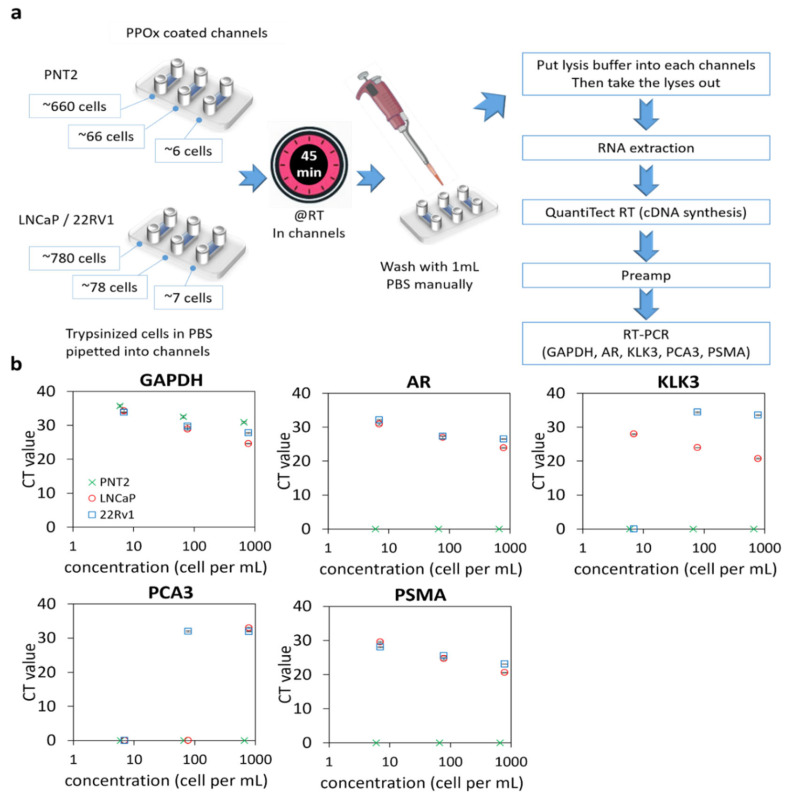
qRT-PCR of prostate cells captured in PPOx channels. (**a**) experiment schematic. RT, room temperature. (**b**) gene expression levels (GAPDH, AR, KLK3, PCA3, and PSMA) for normal prostate PNT2 and prostate cancer LNCaP and 22Rv1 cells spiked in different cell numbers.

We used qRT-PCR for prostate markers to assess the sensitivity of detection of our microfluidics platform using low numbers of cultured cells. For the three cell types (normal prostate PNT2 and two PCa LNCaP and 22Rv1), decreasing numbers of cells were loaded into the PPOx-coated microchannels, which allow non-specific cell capture. qRT-PCR analysis was conducted on the cells bound to the microchannels to detect mRNAs associated with prostatic tissues, androgen receptor (AR), and prostate-specific antigen (kallikrein-related peptidase 3 or KLK3), as well as prostate tumor-derived genes prostate cancer antigen 3 (PCA3) and PSMA (Figure 2a). PCa-specific biomarkers PCA3 and PSMA were tested to confirm the nature of cancerous cells in the sample. The consistent expression of glyceraldehyde 3- dehydrogenase (GAPDH) in most cell types is well documented [44] and was therefore used as an internal control to normalize the specific gene expressions in all samples. The measured CT values for the different cell numbers are shown in Figure 2b (and Table S1) for the three cell types. As expected, the overall average CT values increased with lower cell numbers.

Except for the housekeeping gene GAPDH, normal PNT2 cells did not generate any signal for any of the targeted genes for all cell concentrations tested. In contrast, both prostate-related (AR and KLK3) and tumor-derived (PCA3 and PSMA) genes were successfully detected in 780 LNCaP or 22Rv1 and 78 22Rv1 cells. However, PCA3 was not detectable using a lower number of LNCaP cells. For 22Rv1, no amplification was observed for PCA3 and KLK3 when only seven cells were loaded in the microchannels. In contrast, PSMA was detected in all concentrations of cancer cells, indicating that this biomarker is a suitable candidate for confirming the cancerous nature of rare cells captured in the microchannels. The assay detection limit was determined to be 6 PNT2 and 7 LNCaP/22Rv1 cells, respectively, based on the presence of any genes tested. The high sensitivity of this assay will be essential for detecting the low quantity of exfoliated cells in urine.

### 3.3. Cellularity of Patient Samples and Genomic Signatures

To determine whether prostate cancer cells were detectable in urine prior to DRE, voided urine specimen collected from 31 patients (Table 1) without prior DRE were tested for GAPDH, KLK3, AR, PCA3, and PSMA via qRT-PCR. In this experiment, 1, 3, or 5 mL of urine samples were used. KLK3 and AR genes were used as markers of prostatic origin to verify the presence of prostate cells or prostatic materials in the urine samples [20,45].

**Figure 3:**
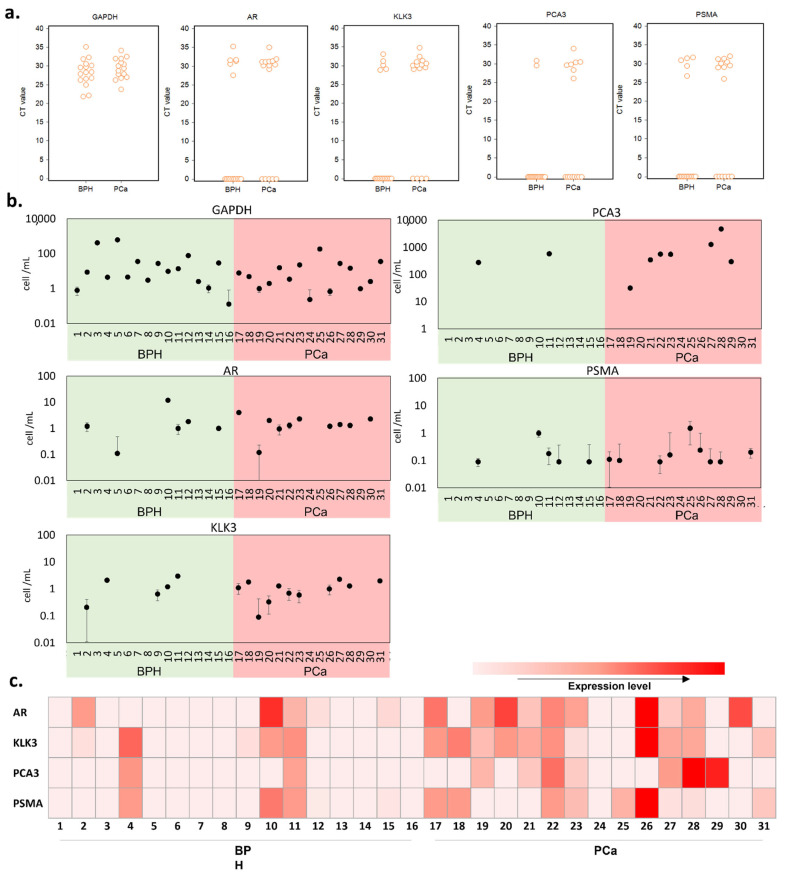
qRT-PCR analysis in BPH and PCa patient urine samples. (**a**) interactive CT value dot plots for the five genes investigated. (**b**) equivalent cell number in the urine sample, as calculated from the spiked-in calibration curve of each gene tested (Figure S1). (**c**) a heatmap representing the various gene expression levels after normalization with GAPDH.

The mRNA expression of each tested gene was quantified by qRT-PCR in the RNA samples extracted from the urinary sediments. The results were first examined in terms of the presence or absence of gene expression in a 1 mL urine sample (Figure 3a). GADPH results indicate that cells were present in all urine samples, with similar levels in benign prostatic hyperplasia (BPH) and PCa samples. Cells expressing any prostate-related genes were present in 70.97% of samples suggesting only 29.03% of cells (expressing GAPDH only) were not of prostate origin, with a higher percentage of prostate cells in the PCa samples compared with BPH (KLK3: 11/15 in PCa patient and 5/16 in BPH patient; AR: 10/15 in PCa patient and 6/16 in BPH patient). PCA3 and PSMA are overexpressed in prostate cancer; thus, they were used as PCa-specific biomarkers in our test [46]. These PCa-specific markers were detected in a greater number of patient samples from the PCa patient group compared with the BPH group (PCA3: 7/15 in PCa patient and 2/16 in BPH patient; PSMA: 9/15 in PCa patient and 5/16 in BPH patient), suggesting PCa samples contain higher numbers of PCa cells. It is worth noting here that PSMA is also expressed on the membrane of other cancers [47] and can be seen in benign brain, kidneys, small intestine, and prostate cells, though in much lesser amounts [48].

Next, we evaluated the cell number per mL of urine samples using the spiked-in calibration curves (Figure 3b and Table S4). To establish the calibration curves, we used filtered BPH patient urine and spiked it with a fixed number of LNCaP cells in a serial dilution. We noted that the CT measured from the same number of cells differed between genes; therefore, the number of cells was estimated for each gene. There was no significant difference in the number of prostate cells detected per mL between BPH and PCa patients, based on the expression levels of all tested genes.

After normalization with GAPDH, we used the prostate-related mRNA expression levels in comparison with the pathological diagnosis. As shown in Figure 3c, altogether, we detected various prostate-related gene signals in 14 (93.33%) of the 15 PCa patients and 7 (43.75%) of the 16 BPH patients. We found that two BPH patients (4 and 11) were positive for both PCA3 and PSMA, while one BPH patient (10) was positive for PSMA. Based on the overall expression of the markers tested, these results confirm the presence of prostate cells in urine without prior DRE. However, it also shows the limited number of prostate cells that can be detected in urine. Moreover, there is not necessarily more cells of prostatic origin in PCa patients. Adding a pre-selective step aiming to enrich the population of PCa cells could help overcome this challenge and enable the detection of PCa in voided urine in the absence of DRE. To test this hypothesis, we next investigated the performance of the selective immunocapture microfluidic platform in patient urine samples.

### 3.4. Selective Cell Capture

A total of 50 urine samples from male patients were tested in the microfluidic device. In the patient cohort, 21 had benign prostatic hyperplasia, and 29 were diagnosed with prostate cancer. The clinical information is provided in Table S2. The device results were compared with histopathology as a gold standard. For each patient sample, a suspension was prepared from the sediment of 2.5 mL of urine containing HAL (50 µM) and nuclear red stain. HAL was used to induce cancer-specific PpIX fluorescence [37,49] and the nuclear stain to distinguish cells from other debris and artifacts that could be present in the sample. The suspension was loaded into the three types of microfluidic channels (PPOx, anti-PSMA test channel, and block negative control) and left to react for 45 min before rinsing with PBS to dislodge loosely bound cells.

**Figure 4:**
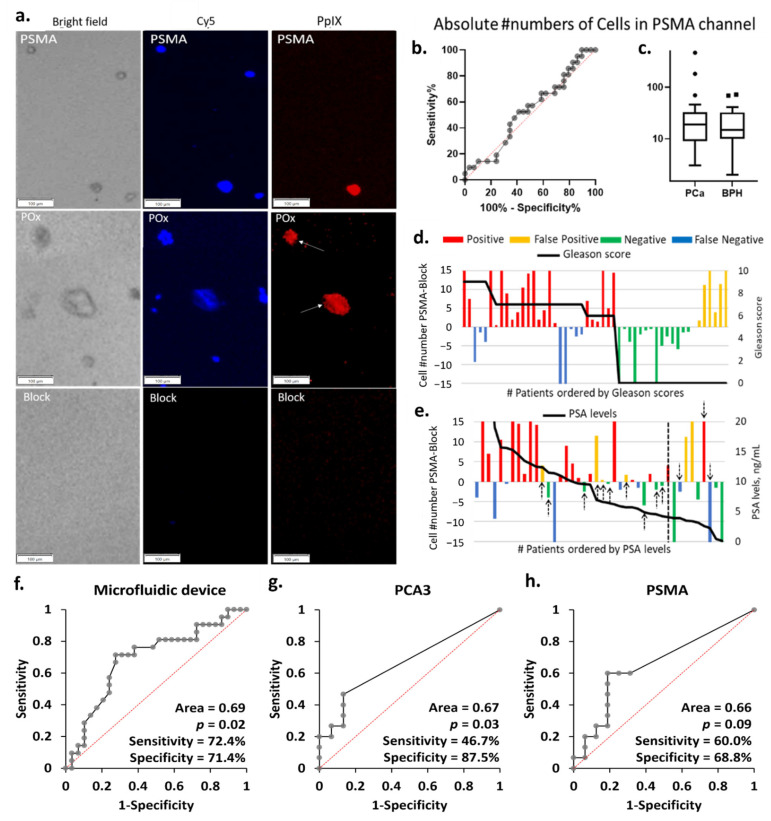
(**a**) Representative PpIX fluorescent images of the block, PSMA, and PPOx microchannels after rinse. Little to no cells seen in the block channel. Conversely, more non-specific cells and debris were captured in the PPOx channel (white arrows). Scale bars represent 100 µm. (**b**) ROC curve and (**c**) box plots of the absolute numbers of cells captured in the PSMA channels for PCA (*n* = 29) and BPH (*n* = 21) patients groups, ▪ and • represents outliers; absolute number of PpIX fluorescence cell as a function of the patient (**d**) Gleason scores and (**e**) PSA levels. Receiver operating characteristic (ROC) curves for PCa diagnosis of patient urine samples in (**f**) microfluidic device, (**g**) urinary PCA3, and (**h**) PSMA by qRT-PCR.

Zoomed-in micrographs of the cell population present in the microchannels post rinse are shown in Figure 4a and zoomed out the field of view in Figure S2. Objects displaying colocalized nuclear red and PpIX fluorescence were recorded as suspicious cells. For all specimen investigated, cells were captured in the microchannels, even in the blocked negative control. This is attributed to residual non-specific binding, as previously noted in the cell line experiments.

As a result, the average of the absolute numbers of suspicious cells bound to the anti-PSMA channels did not correlate with the patient clinical diagnosis, as shown in Figure 4b,c. In order to correct for non-specific binding and variation in overall sample cellularity, the average number of cells bound in the negative control block channel was set as background. The test was counted as positive when the number of cells specifically bound to the anti-PSMA channels was greater than that in the blocked channel and negative otherwise. These cells numbers are reported in Figure 4d,e as a function of the patient Gleason scores and PSA levels. Following this correction for background non-specific binding, the microfluidic chip test reached 72% sensitivity and 71% specificity, as summarized in Table S3. The eight false-negative results are distributed across the different Gleason scores, not showing a particular correlation to the cancer stages. The microfluidic device results largely correlated with PSA levels, with all but one negative result corresponding to PSA level below 10 ng mL^−1^. It is worth noting that using PSA levels with a cut-off value of 4 ng mL^−1^ for initial referral to biopsy would, in this cohort, result in 3 false negative and 10 false positive (black arrows in Figure 4e), 8 of which had PSA levels below 10 ng mL^−1^. If cases with PSA levels below 10 ng mL^−1^ were only considered positive when the microfluidic device also returned a positive result, then only 5 unnecessary biopsies would be conducted instead of 10.

### 3.5. Comparison of the Diagnostic Performance of the Microfluidic Device with qRT-PCR

Using the histopathological diagnosis as gold standard, the performance of the microfluidic device was evaluated by ROC analysis and compared to the qRT-PCR results using a contingency table (Figure 4f–h and Table S3). The outcome of the contingency table was assessed based on positive and negative results. The microfluidic device was capable of distinguishing PCa from BPH with sensitivity of 72.4% and specificity of 71.4%. We then identified the diagnostic accuracy of urinary PCA3 and/or PSMA mRNA expression detected by qRT-PCR. The urinary PCA3 result showed a low sensitivity of 46.7% and specificity of 87.5%, confirming the observation made in the cell line experiment. The urinary PSMA displayed better sensitivity (60.0%) but lower specificity (68.8%) when compared with both urinary PCA3 and the cell capture approach which returned the best PPV, and overall diagnostic potential (*p* = 0.02). The diagnostic performance was further validated by calculating different combinations detected in urine samples. The highest sensitivity (80.0%) with lower specificity (68.8%) was obtained if either PCA3 or PSMA were detected, whilst the lowest sensitivity (26.7%) with highest specificity (87.5%) was measured if both PCA3 and PSMA were expressed. Further validation was performed by ROC curve analysis. The difference in absolute numbers of PpIX fluorescent cells between the test and block channels in microfluidic device and the normalized mRNA expression levels (PCA3, or PSMA) measured in qRT-PCR were used. The diagnostic accuracy of ROC analysis generated similar results to the contingency table (Figure S3 and Table S5). Overall, these results demonstrated the microfluidic device had clinical potential for detecting PCa in urine samples.

### 3.6. Captured Cells Compared to Biopsy Outcomes and Voided Urine

**Figure 5:**
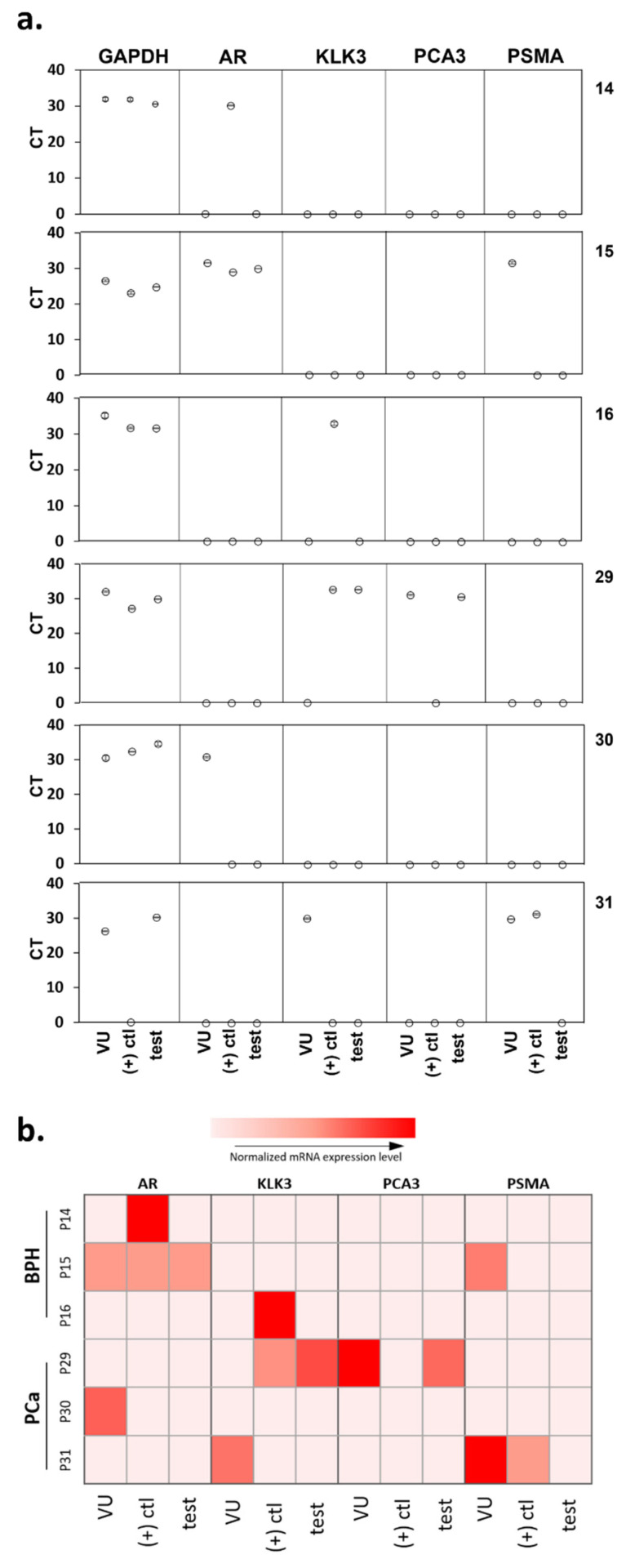
qRT-PCR analysis showing (**a**) the actual CT value measured. (**b**) a heatmap representing the various gene expression levels after normalization with GAPDH for the voided urine samples and the urinary cells captured in both the positive control and test microfluidic channels. VU, voided urine; (+) ctl, positive control channel; test, test channel.

qRT-PCR was used to investigate the occurrence of false-negative results in the microfluidic device. PCR was conducted on the cellular content of the positive control, and test microchannels for six of the urine samples run through the microfluidic device, as well as directly on the corresponding sediment of 5 mL of urine. Three patients were diagnosed by histopathology to have BPH (14, 15, and 16), and three had PCa (29, 30, and 31), two of which returned a false-negative result (30 and 31) with the microfluidic device. Among these six patients, the sensitivity and specificity of the device were 33.3% and 100%, respectively (Figure 5).

Higher expression of tumor-derived RNA biomarkers (PCA3 and PSMA) was observed in the PCa patients compared to those of BPH patients, in agreement with findings above (Figure 3). In fact, no expression of any tumor-derived markers was detected in the lysate of BPH patients 14 and 16 confirming the absence of PCa cells in these samples (Figure 5). These data verify the specificity of the microfluidics device in capturing PCa cells.

However, there were some inconsistencies observed. In PCa sample 31, PCA3 was not detected, and PSMA was detected in the voided urine sample and the positive control channel but absent in the test channel lysate. In this case, the absence of PSMA-positive cells cannot explain the negative results obtained with the device. Interestingly, the expression levels within the voided urine sediment often did not correlate with lysate extracted from the captured cells, despite the fact that the latter originates from the concentrate of a smaller volume of urine. Examination of a larger number of patient samples may help resolve some of these inconsistencies and provide more comprehensive validation of the microfluidics platform.

## 4. Discussion

The microfluidic device tested in this study has been developed with the aim of capturing PCa cells present in the voided urine of PCa patients without prior DRE. This would represent a significant improvement on current protocols of PCa diagnosis, which rely on insensitive (PSA) or more invasive (DRE) procedures. PCR analysis was used to test this hypothesis on the sediment of 1 to 5 mL of voided urine sample. The results indicate that cells were present in all urine samples, some of which were of prostatic origin based on their expression of KLK3 and AR (Figure S1 and Table S3). These biomarkers were chosen because, in prostate cells, AR regulates KLK3 mRNA (which produces PSA protein). Correlated expression of AR and KLK3 is generally a suitable indicator of prostatic origin; however, these markers are not infallible. For instance, the expression of AR by PNT2 cells remained controversial. It has been previously described as AR-negative [50,51] and sometimes AR-positive [52]. In our experiments, PNT2 did not express AR nor KLK3. Thus, AR-negative prostate cells may express low levels of KLK3 and be difficult to detect. It is also worth noting that many cells that are not of prostatic origin can express AR mRNA [53]. This means that in healthy patient samples, expression of AR in the absence of other prostate-related gene expressions can indicate the presence of cells that are not of prostatic origin. Only one case was recorded in the cohort of BPH investigated here (Table S2). In PCa patients, however, the correlation between AR and KLK3 can be even less straightforward when the patient is treated with androgen deprivation therapy (ADT). In which case, the AR mRNA can often increase as KLK3 mRNA decreases [54]. For PCa patients, prostate cancer cells may be present despite the absence of KLK3 mRNA. This appears to be the case for three of the PCa specimens investigated here.

The nature of prostatic-origin and -cancerous cells in patient urine samples and lysate of urinary cells captured were cross-validated and further confirmed by qRT-PCR. To assess the presence of PCa cells in the urine sediment, the PCR test targeted PCA3, a prostate tumor-derived gene and PSMA that is highly expressed in prostate cancer tissues. The results of the differential gene expression test indicated higher expression of PCA3 and PSMA in the PCa patient group, suggesting that cancer cells could be detected in voided urine without DRE. However, PCA3 exhibited relatively low sensitivity, in suitable agreement with previous reports [55,56]. Altogether the PCR tests indicated that only a small number of PCa cells could be expected in the voided urine, which could explain the modest sensitivity of the microfluidic device. While initial tests with cultured cell lines proved that the device performed well even for a ratio of cancer to healthy cells of 1:50, it only achieved 72% sensitivity in patient samples. qRT-PCR of the lysate of cells captured in the microchannels indicated the presence of cells expressing AR and/or KLK3 but not necessarily that of tumor-derived PCA3 and PSMA genes. Since the effectiveness of the capture is based on the presence of PSMA on the surface of PCa cells, the microfluidic device may fail to detect PCa and return false-negative results in two instances: absence of PCa cells and/or altered PSMA expression on the PCa cells present. False-positive results, on the other hand, may be due to the expression of PSMA in normal prostate tissues or other normal human tissues and cancers [32,57], as well as from the non-specific binding of a benign cell type that intrinsically fluoresces at the same wavelength as PpIX.

Comparable studies applying whole cancer cell capture methods to clinical urine samples of prostate cancer patients are rare but report similar results. Specifically, Rzhevskiy and colleagues achieved 79% efficiency via immunocytochemistry with a single biomarker in a spiral microfluidic device [58]. Our approach differs from theirs in using dual techniques of specific immunocapture with photodynamic detection within one assay. While the results presented in this work are promising, future development will involve combining multiple biomarkers to target more aggressive types of cancer cells in an attempt to improve the sensitivity and specificity of the microfluidic device. The versatility of our technology would easily accommodate such extensions. A more accurate detection device will help to reduce overdiagnosis, which can lead to unnecessary harmful surgeries and treatments.

Altogether, substantial modifications would be necessary to improve the diagnostic precision of this method for prostate cancer detection. First, we did not apply any preservative reagent for urine collection and storage, which might affect the sample cellular material quality. However, those reagents may contain proteins that could interfere with the detection if they were to non-specifically bind to the test or control channels. For this reason, the experimental protocol of the test has been carefully designed to limit the non-specific binding of objects that do not express cancer-specific antibodies. Non-specific binding to the biofunctionalized microchannels is kept to a minimum using appropriate blocking and rinsing protocols, as previously demonstrated [21,59]. Specifically, the skim milk blocking agent prevents protein present in the biological fluid from binding to the PPOx-coated substrate and obstructing the antibody binding sites. Second, although our data (Figure S4) suggest that the initial sample volume has no impact on gene expression level (1 mL vs. 3/5 mL), we believe that a larger sample volume could increase the cell yields. Lastly, both sensitivity and selectivity could be improved by combining more biomarkers than just PSMA and PpIX. Therefore, further modification of the method and the device comprising additional specific cancer antibodies could achieve diagnostic sensitivity and specificity for clinical utility.

## 5. Conclusions

In summary, we have developed a microfluidic device for PCa detection in liquid biopsies with a low cancer cell detection limit. We demonstrated the presence of prostate biomarker expression in cultured cells and PCa patient urine samples captured by this device. Comparisons of the device with qPCR of prostate biomarkers indicated comparable sensitivity and specificity of detection. Collectively, these findings indicate that the device had the clinical potential to detect PCa cells from urine in the absence of DRE. Further validation cohort studies are needed to support the clinical utility of the device. We envision that with these and other refinements, this device could potentially be developed for a noninvasive cancer diagnosis.

## Figures and Tables

**Table 1:** Clinical data for the patient cohort investigated via qRT-PCR.

Characteristics	BPH	PCa
Patients, *n* (%)	16 (51.6)	15 (48.4)
Age, median (range)	66 (58–86)	64 (52–73)
PSA diagnosed, *n* (%)		
≤10 ng mL^−1^	11 (68.8)	8 (53.3)
>10 ng mL^−1^	1 (6.3)	6 (40.0)
Unknown	4 (25.0)	1 (6.7)
PSA ng mL^−1^, median (range)	4.4 (0.1–12.4)	9.61 (2.3–466)
Pathological T stage, *n* (%)		
pT2a-c	NA	6 (40.0)
pT3a-c	NA	2 (13.3)
Unknown	NA	7 (46.7)
Gleason score, *n* (%)		
≤6	NA	2 (13.3)
7	NA	8 (53.3)
≥8	NA	5 (33.3)

BPH = benign prostatic hyperplasia; PCa = prostate cancer; PSA = prostate-specific antigen; NA = not available.

## Data Availability

The data are presented within the article and Supplementary Materials.

## Supplementary Materials

The following are available online https://www.mdpi.com/article/10.3390/cancers13215544/s1. Details of materials and methods: details of targeted genes used in qRT-PCR, Figure S1: Spiked-in calibration curves. (a) Schematic diagram of the experimental process. Created with Biorender.com; (b) Equivalent cell number calibration curves and protocol used to estimate the equivalent cell number in patient urine samples, Figure S2: microscopic images showing the PpIX fluorescence in prostate cancer LNCaP and in mix cell suspension after treated with HAL compared to normal prostate PNT2 cells. Scale bars represent 100 µm, magnification 10×, Figure S3: a) Box plot and b) ROC curves of normalized mRNA expression in voided urine samples (*n* = 31). Based on the 2^−ΔCT^ values, Figure S4: CT values measured using 1 and 3/5 mL of urine for qRT-PCR. Red bars denotes “PCa patient” (*n* = 7) and gray bars denotes “BPH patient” (*n* = 7). Experiment performed on the same day for each patient, in triplicate, Table S1: CT values measured from a different number of prostate cells captured in PPOx channels, Table S2: Summary of cohort patient clinical data, urinalysis, and microfluidic device results, Table S3: Diagnostic performance of the microfluidic device (*n* = 50) compared with prostate tumor-derived urinary PCA3 and/or PSMA (*n* = 31), Table S4: Patients clinical information and estimated cell number according to calibration curves, Table S5: ROC curve analysis results.
