# Supplementary material for: Selective Microfluidic Capture and Detection of Prostate Cancer Cells from Urine without Digital Rectal Examination
Source: Cancers (Basel). 2021 Nov 4;13(21):5544. doi: 10.3390/cancers13215544 (PMC8583121; doi:10.3390/cancers13215544)
Supplement: Supplementary file 1 [file cancers-13-05544-s001.zip › cancers-1406344-supplementary.pdf]

cancers

MDPI

Supplementary materials: Selective Microfluidic Capture and Detection of Prostate Cancer Cells from Urine without Digital Rectal Examination

Kit Man Chan, Jonathan M. Gleadle, Philip A. Gregory, Caroline A. Phillips, Hanieh Safizadeh Shirazi, Amelia Whiteley, Jordan Li, Krasimir Vasilev and Melanie MacGregor

Supplementary materials and methods

1. Cell culture

Human normal prostate epithelium PNT2 (cat no. 95012613) and prostate carcinoma LNCaP clone FGC (cat no. 89110211) were purchased from CellBank Australia (Westmead, NSW, Australia). The prostate carcinoma epithelial 22Rv1 cell line was kindly gifted by Dr. Luke Selth, University of Adelaide. Cells were cultured in RPMI-1640 medium (Sigma-Aldrich, Castle Hill, Australia) supplied with 10% fetal calf serum and 1% (v/v) penicillin/streptomycin (Thermo Fisher Scientific, Scoresby, Australia) and grown at 37 °C in 5% CO_2 incubator.

2. Patient sample

The study consisted of 50 men who were enrolled at Flinders Medical Centre and Noarlunga Hospital (Adelaide, Australia) between July 2019 and August 2020 with suspicion of prostate cancer. The diagnosis in each case was confirmed by histopathology. Of the 50 patients, 29 patients were diagnosed with PCa and 21 with benign prostatic hyperplasia. This study was approved by the Southern Adelaide Clinical Human Research Ethics Committee (approval number HREC/19/SAC/33). Written informed consent was obtained from each patient. Urine samples were collected without previous prostate massage. Clinical pathologic (Histopathology, Gleason score, tumor stage, PSA level) and urinalysis test results (pH, presence of proteins and blood cells) were recorded for review.

3. Preparation of microfluidic device

The three PPOx-coated microchannels of each PMMA chip were set as test, negative control and positive control respectively. The positive control channel was used as is, with the biocompatible PPOx surface allowing the non-specific attachment of all cell types. The test channel was biofunctionalized with 60 µL of anti-PSMA antibodies (10 µg mL^{-1}). Then, 1 mg mL^{-1} skim milk solution, acting as a block solution, was added to both the test and the negative control channels for 45 min. The slides were rinsed with phosphate buffered saline (PBS) three times and fresh PBS was added afterwards.[1] Following this procedure, the PPOx coating present in the negative control channel is blocked by the skim milk proteins which prevents the non-specific binding of any cell types. In the test channel, the skim milk blocking step is used to limit non-specific cell binding from happening in between the surface bound PSMA antibodies.

4. Cell capture efficiency on the micofluidic platform

In order to efficiently capture PCa cells from urine, we optimized our previously designed microfluidics device.[1] to include specific immunocapture capability. Normal prostate epithelial PNT2 cells and prostate cancer LNCaP cells were trypsinized. The cell suspensions were then centrifuged at 1200 rpm for 5 min, the supernatant removed and the cell pellet resuspended in fresh PBS to the targeted concentration range around 2 x 10^5 cells mL^{-1}. Cells were treated with 0.5 µM Nuclear-Red LCS1 (AAT Bioquest, CA 94085,

USA) and 50 μM Hexaminolevulinate (HAL) hydrochloride (Sigma-Aldrich, NSW, Australia)[2] for 1 h at room temperature in the dark. Then PNT2 and LNCaP cells were mixed together and adjusted with varying ratios (1:1; 1:10 and 1:50). 60 μL of the mixed cell suspension were added to each microchannels (test, negative control, positive control) for capture and imaging.

Two prostate cancer cell lines LNCaP and 22Rv1 were used to determine the limit of detection of PCR analysis conducted on cells captured in the microchannels. Prostate normal epithelial PNT2 cell line was applied as a negative control. Cell suspensions with 7, 78, 780 LNCaP / 22Rv1 cells and 6, 60, 660 PNT2 cells in 60 μL PBS were prepared and then dispensed into PPOx microchannels. The cells captured in the channels after imaging were processed to RNA extraction as described in the next section.

5. RNA extraction from cultured cells spiked in experiment and patient urine samples

For spiked-in calibration curves purpose (see Supplementary information and Figure S1 for more details) and to define the limit of detection for low prostate cancer cell numbers in patient urine, cultured cells (concentration range between 2 to 90000 cells) were spiked into 1 mL of filtered urine from a non-cancer patient urine sample.

For actual testing of patient urine sample by qRT-PCR, different volumes (1, 3, 5 mL) of urine were collected. In both cases, the urine samples were centrifuged at 1200 rpm for 5 minutes. The supernatant was discarded and the cell pellets was resuspended. Total RNA was extracted from the cells with the RNeasy Plus Micro Kit (Qiagen, VIC, Australia) according to the manufacturer's protocol.

6. RNA extraction from cultured cells and patient urine samples in microchannels

Cultured cells and cells present in the patient urine samples were captured in the microchannels as described in "Cell capture efficiency on the microfluidic platform". The total RNA was extracted using a RNeasy Plus Micro Kit (Qiagen, VIC, Australia). 75 μL of RLT buffer was added to each of the microchannels to lyse the captured cells out. The lysate was transferred to a microcentrifuge tube and the RNA extraction was performed with the kit according to the manufacturer's protocol.

7. Targeted genes tested in qRT-PCR

The following TaqMan primer/probes from Thermo Fisher Scientific were used: androgen receptor (AR) (Hs00171172_m1), kallikrein related peptidase 3 (KLK3) (Hs02576345_m1), prostate cancer associated 3 (PCA3) (Hs01371939_g1), prostate-specific membrane antigen (PSMA) (Hs00379515_m1) and glyceraldehyde-3-phosphate dehydrogenase (GAPDH) (Hs02786624_g1). The preamplification was applied on a MiniAmp Thermal Cycler (Applied Biosystems). Thermal protocol conducted as: 95°C for 10 minutes, followed by 14 cycles of amplification (95°C for 15 seconds and 60°C for 4 minutes) and 99°C for 10 minutes. Quantitative RT-PCR was performed in a LightCycler 480 Instrument II (Roche Diagnostics, NSW, Australia). The same primer/probe set were used in qRT-PCR. Each 10 μL sample contained 2 μL of preamplified and diluted cDNA, 2X TaqMan Universal Master Mix II with UNG and each 20X primer/probe. The qRT-PCR was run at 50°C for 2 minutes, followed by 95°C for 10 minutes and 50 cycles of amplification at 95°C for 15 seconds and 60°C for 1 minute. Prostate in origin (AR & KLK3) and tumor-derived (PCA3 & PSMA) biomarkers were tested. GAPDH was used as the housekeeping gene to evaluate the cDNA quality.

PSMA: encodes a type II transmembrane glycoprotein belonging to the M28 peptidase family. This protein is expressed in a number of tissues such as prostate. In the prostate the protein is upregulated in cancerous cells and is used as an effective diagnostic and prognostic indicator of prostate cancer.

PCA3: This gene produces a spliced, long non-coding RNA that is highly overexpressed in most types of prostate cancer cells and is used as a specific biomarker for PCa

detection. In prostate cancer derived cells, overexpression of PCA induced downregulation of prune2, leading to decreased cell proliferation. Regulation of this gene appears to be sensitive to androgen-receptor activation, a molecular signature of prostate cancer.

KLK3: Growing evidence suggests that many kallikreins are implicated in carcinogenesis and some have potential as novel cancer and other disease biomarkers. This gene is one of the fifteen kallikrein subfamily members located in a cluster on chromosome 19. Its protein product is a protease present in seminal plasma. It is thought to function normally in the liquefaction of seminal coagulum, presumably by hydrolysis of the high molecular mass seminal vesicle protein. Serum level of this protein, called PSA in the clinical setting, is useful in the diagnosis and monitoring of prostatic carcinoma.

AR: The androgen receptor gene is more than 90 kb long and codes for a protein that has 3 major functional domains: the N-terminal domain, DNA-binding domain, and androgen-binding domain. The protein functions as a steroid-hormone activated transcription factor. Mutations in this gene are also associated with complete androgen insensitivity (CAIS).

GAPDH: This gene encodes a member of the glyceraldehyde-3-phosphate dehydrogenase protein family. The product of this gene catalyzes an important energy-yielding step in carbohydrate metabolism, the reversible oxidative phosphorylation of glyceraldehyde-3-phosphate in the presence of inorganic phosphate and nicotinamide adenine dinucleotide (NAD). This protein has assigned a variety of additional functions including nitrosylation of nuclear proteins, the regulation of mRNA stability.

8. Spiked-in calibration curves:

Trypsinized prostate cancer LNCaP cells were serial diluted to 2×10^5 cells mL^{-1} with PBS. The cell solution was dispensed into 1 mL of filtered (0.22 μm) Benign prostatic hyperplasia (BPH) patient urine sample with final cell number ranging from 9×10^4 cells mL^{-1} to 2×10^0 cells mL^{-1}. The serial diluted cell solutions were used to build a calibration curve. RNA extraction, cDNA synthesis, preamplification and qRT-PCR were conducted as described as above. Cycle threshold values were measured to obtain a spiked-in calibration curve for each tested gene. The calibration curves were used to evaluate the "equivalent" cell number per mL of patient urine samples (Figure S1).

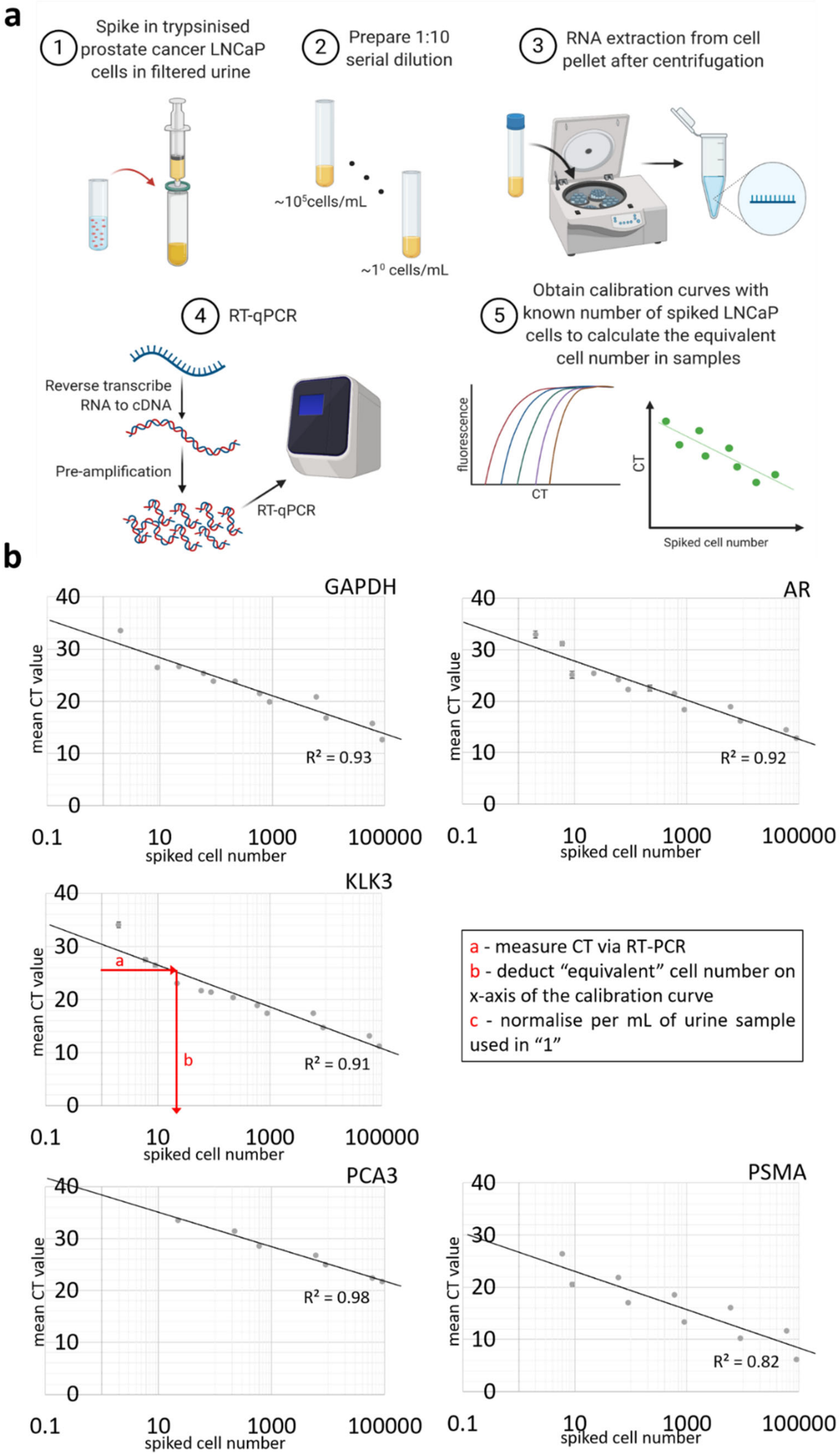

Figure S1. Spiked-in calibration curves. a) Schematic diagram of the experimental process. Created with Biorender.com; b) Equivalent cell number calibration curves and protocol used to estimate the equivalent cell number in patient urine samples.

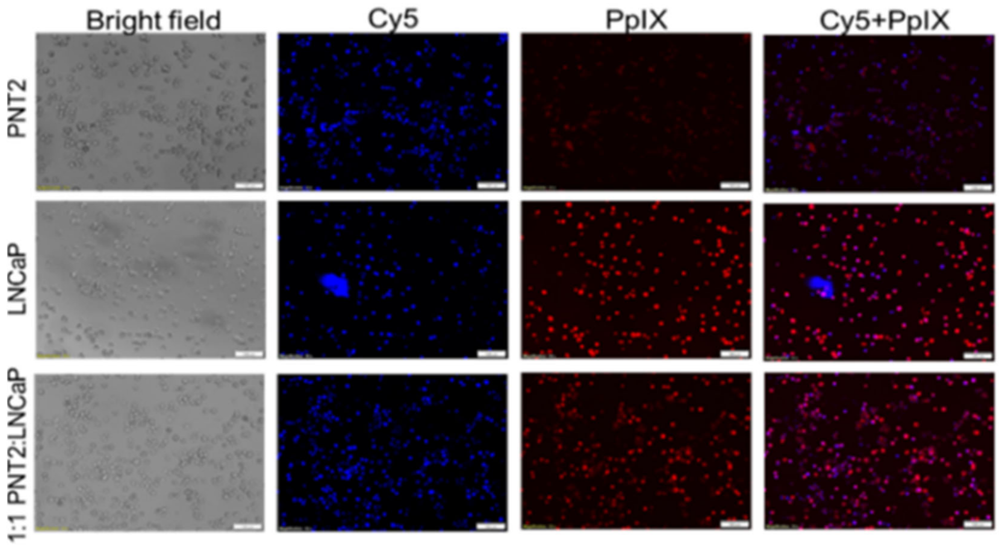

Figure S2. microscopic images showing the PpIX fluorescence in prostate cancer LNCaP and in mix cell suspension after treated with HAL compared to normal prostate PNT2 cells. Scale bars represent 100 µm, magnification 10X.

Table S1. CT values measured from different number of prostate cells captured in PPOx channels.

**Cell lines**	number of cells loaded	Genes tested				
		GAPDH	AR	KLK3	PCA3	PSMA
**PNT2**	660	30.9	0	0	0	0
	66	32.49	0	0	0	0
	6	35.69	0	0	0	0
LNCaP	780	24.61	23.91	20.74	32.97	20.62
	78	28.87	26.96	23.94	0	24.6
	7	34.21	30.88	27.95	0	29.53
22Rv1	780	27.74	26.49	33.56	31.89	23.05
	78	29.72	27.33	34.41	31.95	25.51
	7	33.79	32.09	0	0	28.03

Table S2. Summary of cohort patient clinical data, urinalysis and microfluidic device results.

Patient [a)]	Age	Histo-pathology	Gleason score	Stage [b)]	PSA (ng mL^{-1})	Urinalysis [c)]									Device
						pH	NIT	PRO	GLU	KET	BIL	ERY	LEU	Blood	
1	64	BPH	NA	NA	0.12	6.5		1+						moderate	Negative
2	66	BPH	NA	NA	NA	5		trace						moderate	Negative
3	77	BPH	NA	NA	6.65	7		1+						moderate	Negative
4		BPH	NA	NA	5.72	5									False positive
5	86	BPH	NA	NA	3.91	5	+	2+					2+	large	Negative
6		BPH	NA	NA	3.5	6		trace					trace	moderate	False positive
7	72	BPH	NA	NA	NA	6		trace							Negative
8		BPH	NA	NA	NA	7		1+						large	False positive
9	58	BPH	NA	NA	4.56	6		1+		1+			1+	large	Negative
10	66	BPH	NA	NA	4.25	6		trace							Negative
11	69	BPH	NA	NA	6.48	5							trace		Negative
12	64	BPH	NA	NA	NA	5	+	4+					2+	large	Negative
13		BPH	NA	NA	12.4	6								trace	False positive
14	59	BPH	NA	NA	3.15	5		1+			1+			moderate	Negative
15	64	BPH	NA	NA	4.86	5		trace					trace	trace	Negative
16	72	BPH	NA	NA	0.41	6		trace			1+				Negative
17		PCa	7	PT2	5.7	6							1+	trace	False negative
18		PCa	7	NA, biopsy only	2.62	5		trace				1+			False negative
19	64	PCa	7	PT3aN0Mx	10.4	6		trace							Positive
20	53	PCa	7	PT2cN0	9.55	7		1+						moderate	Positive
21	73	PCa	9	NA, biopsy only	6.19	5		trace						moderate	Positive
22	65	PCa	7	PT2cN0	15.7	6									Positive
23	64	PCa	6	NA, biopsy only	30.7	7		1+					2+		Positive
24		PCa	7	NA, biopsy only	2.26	5		trace						large	False negative
25	68	PCa	7	NA, biopsy only	13	6		1+			1+		1+	large	Positive
26	52	PCa	7	PT3bNxMx	9.67	6									Positive
27		PCa	9	PT2N0	19	6		trace	4+					trace	False negative
28	60	PCa	6	NA, biopsy only	4.68	6									Positive
29	59	PCa	9	PT2N0	NA	6		trace			1+				Positive
30	56	PCa	9	PT2cN0	5.5	6.5								trace	False negative

31	71	PCa	9	NA, biopsy only	466	6.5		trace			1+			False negative
32		BPH	NA	NA	11.5	6		1+				trace	large	Negative
33		BPH	NA	NA	NA	5		1+		1+				Negative
34		BPH	NA	NA	9.63	6.5		trace			1+			Negative
35	66	BPH	NA	NA	6.96	7								False positive
36	86	BPH	NA	NA	3.4	6	+	2+				2+	moderate	False positive
37		PCa	6	NA, biopsy only	NA	8		1+						Positive
38		PCa	7	PT2NX	4.1	7								Positive
39		PCa	7	NA, biopsy only	12.5	5		trace			1+		trace	Positive
40		PCa	8	NA, biopsy only	324	5		2+				trace	moderate	Positive
41		PCa	7	NA, biopsy only	11.43	5			4+					False negative
42		PCa	7	NA, biopsy only	13.5	7		1+		trace	1+		moderate	Positive
43		PCa	7	NA, biopsy only	15.6	5		2+						Positive
44		PCa	7	NA, biopsy only	10	6		trace					trace	Positive
45		PCa	7	PT2N0	NA	5		trace				trace		Positive
46		PCa	6	NA, biopsy only	11	6	+	trace				2+	trace	Positive
47	63	PCa	7	PT2cN0	3.87	6.5								Positive
48	72	PCa	6	NA, biopsy only	15.3	6.5	+	3+				2+	large	Positive
49	65	PCa	7	NA, biopsy only	5.9	5								Positive
50	72	PCa	NA	NA, biopsy only	14.5	8.5		1+			1+			Positive

BIL = Bilirubin; BPH = benign prostatic hyperplasia; ERY = erythrocyte; GLU = glucose; KET = ketone; LEU = leucocyte; NA = not available; NIT = nitrate; PCa = prostate cancer; PRO = protein; PSA = prostate-specific antigen. a) The quantitative reverse-transcriptase polymerase chain reaction (qRT-PCR) result of patient 1 to 31 was discussed in "Cellularity of patient samples and genomic signatures" section; b) the stage of cancer is determined by the Tumour-Node-Metastasis (TNM) system; c) urinalysis is evaluated by Dipstick test.

Table S3. Diagnostic performance of the microfluidic device ($n = 50$) compared with prostate tumor-derived urinary PCA3 and/or PSMA ($n = 31$).

a)	Microfluidic device			Urinary PCA3 b)			Urinary PSMA b)			Either urinary PCA3 or PSMA b)			Both urinary PCA3 and PSMA b)		
	Posi-	Nega-	Total	Posi-	Nega-	Total	Posi-	Nega-	Total	Posi-	Nega-	Total	Posi-	Nega-	Total
**PCa**	21	8	29	7	8	15	9	6	15	12	3	15	4	11	15
**BPH**	6	15	21	2	14	16	5	1	16	5	11	16	2	14	16

**Total**	27	23	9	22	14	17	17	14	6	25
**Sensitivity (95% CI)**	72.4% (52.8-87.3%)		46.7% (21.3-73.4%)		60.0% (32.3-83.7%)		80.0% (51.9-95.7%)		26.7% (7.8-55.1%)	
**Specificity (95% CI)**	71.4% (47.8-88.7%)		87.5% (61.7-98.4%)		68.8% (41.3-89.0%)		68.8% (41.3-89.0%)		87.5% (61.7-98.5%)	
**PPV (95% CI)**	77.8% (63.2-87.7%)		77.8% (46.2-93.5%)		64.3% (43.8-80.6%)		70.6% (52.7-83.8%)		66.7% (29.9-90.4%)	
**NPV (95% CI)**	65.2% (49.5-78.2%)		63.6% (51.3-74.4%)		64.7% (47.6-78.7%)		78.6% (55.8-91.4%)		56.0% (47.1-64.5%)	
**Accuracy (95% CI)**	72.0% (57.5-83.8%)		67.7% (48.6-83.3%)		64.5% (45.4-80.8%)		74.2% (55.4-88.1%)		58.1% (39.1-75.5%)	

NPV = negative predictive value; PPV = positive predictive value. a) test performance was calculated from 2 x 2 contingency tables diagnostic test, outcomes are assessed based on the positive and negative results; b) results are obtained from qRT-PCR.

Table S4. Patients clinical information and estimated cell number according to calibration curves.

	Clinical data					Estimated number of cell/mL				
	histopathology	Age	Gleason Score	Stage	PSA	GAPDH	AR	KLK3	PCA3	PSMA
1	Benign prostatic hyperplasia	64	NA	NA	0.12	0.1<n< 1	0	0	0	0
2		66	NA	NA	NA	1<n<10	1<n<10	0.1<n<1	0	0
3		77	NA	NA	6.65	100<n< 1000	0	0	0	0
4		NA	NA	NA	5.72	1<n<10	0	1<n<10	100<n< 1000	n<0.1
5		86	NA	NA	3.91	100<n< 1000	0.1<n<1	0	0	0
6		NA	NA	NA	3.5	1<n<10	0	0	0	0
7		72	NA	NA	NA	10<n<100	0	0	0	0
8		NA	NA	NA	NA	1<n<10	0	0	0	0
9		58	NA	NA	4.56	10<n<100	0	0.1<n<1	0	0
10		66	NA	NA	4.25	10<n<100	10<n<100	1<n<10	0	1<n<10
11		69	NA	NA	6.48	10<n<100	1<n<10	1<n<10	100<n< 1000	0.1<n<1
12		64	NA	NA	NA	10<n<100	1<n<10	0	0	n<0.1
13		NA	NA	NA	12.4	1<n<10	0	0	0	0
14		59	NA	NA	3.15	1<n<10	0	0	0	0
15		64	NA	NA	4.86	10<n<100	1<n<10	0	0	n<0.1
16		72	NA	NA	0.41	0.1<n<1	0	0	0	0
17	Prostate adenocarcinoma	NA	7	PT2	5.7	1<n<10	1<n<10	1<n<10	0	0.1<n<1
18		NA	7	NA, biopsy only	2.62	1<n<10	0	1<n<10	0	0.1<n<1
19		64	7	PT3aN0Mx	10.4	1<n<10	0.1<n<1	n<0.1	10<n<100	0
20		53	7	PT2cN0	9.55	1<n<10	1<n<10	0.1<n<1	0	0
21		73	9	NA, biopsy only	6.19	10<n<100	0.1<n<1	1<n<10	100<n< 1000	0
22		65	7	PT2cN0	15.7	1<n<10	1<n<10	0.1<n<1	100<n< 1000	n<0.1
23		64	6	NA, biopsy only	30.7	10<n<100	1<n<10	0.1<n<1	100<n< 1000	0.1<n<1
24		NA	7	NA, biopsy only	2.26	0.1<n<1	0	0	0	0
25		68	7	NA, biopsy only	13	100<n< 1000	0	0	0	1<n<10
26		52	7	PT3bNxMx	9.67	0.1<n<1	1<n<10	1<n<10	0	0.1<n<1
27		NA	9	PT2N0	19	10<n<100	1<n<10	1<n<10	1000<n<10000	n<0.1
28		60	6	NA, biopsy only	4.68	10<n<100	1<n<10	1<n<10	1000<n<10000	n<0.1
29		59	9	PT2N0	NA	1<n<10	0	0	100<n< 1000	0
30		56	9	PT2cN0	5.5	1<n<10	1<n<10	0	0	0
31		71	9	NA, biopsy only	466	10<n<100	0	1<n<10	0	0.1<n<1

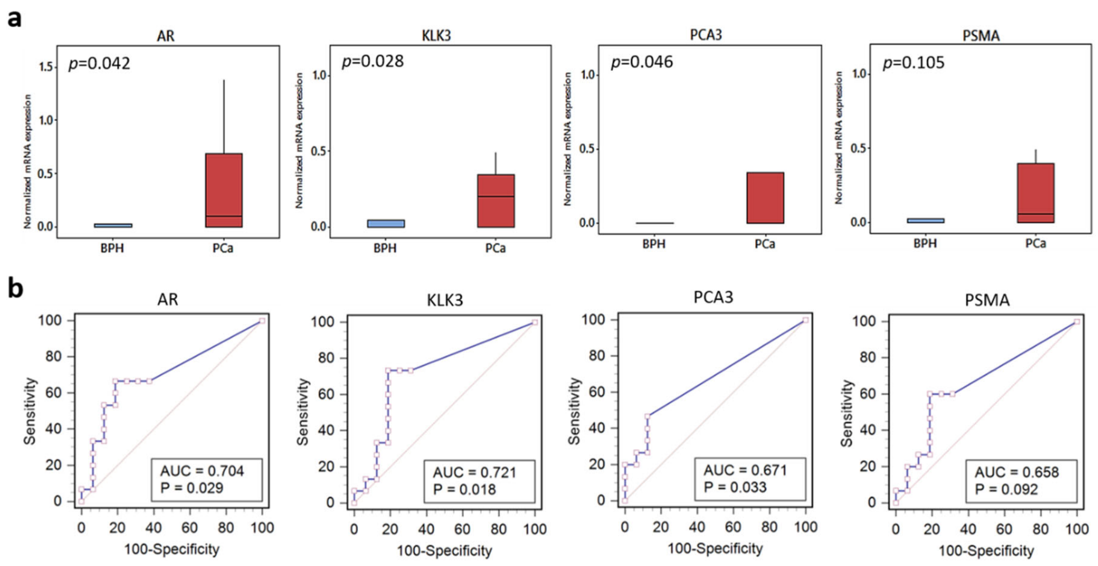

Figure S3. a) Box plot and b) ROC curves of normalized mRNA expression in voided urine samples (*n* = 31). Based on the $2^{-\Delta CT}$ values.

Table S5. ROC curve analysis results.

	**Sensitivity (%)**	**Specificity (%)**	**AUC (95% CI)** [a]	***P***

AR	66.67	81.25	0.704 (0.514-0.854)	0.0289
KLK3	73.33	81.25	0.721 (0.531-0.866)	0.0179
PCA3	46.67	87.50	0.671 (0.480-0.828)	0.0326
PSMA	60.00	81.25	0.658 (0.467-0.818)	0.0921

AUC = area under the ROC curve

[a] binomial exact

The range of AUC from 0.5 (no diagnostic ability) to 1.0 (perfect diagnostic ability). The diagonal (45 degree) line is a reference line of ROC curve and serves as a baseline. The AUC of AR, KLK3 and PCA3 shown significantly different ($p < 0.05$) between BPH and PCa patients. PCA3 had the lowest sensitivity and highest specificity.

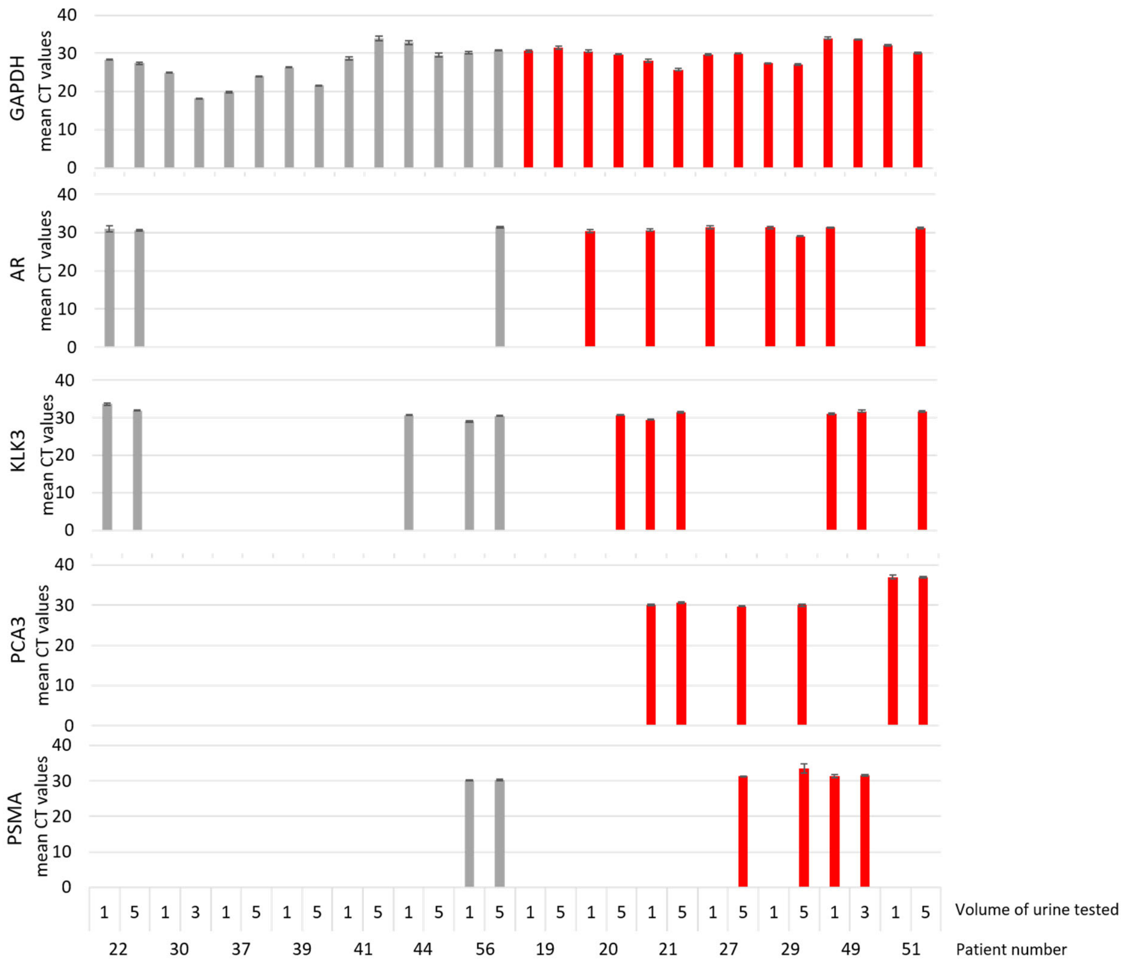

Figure S4. CT values measured using 1 and 3/5 mL of urine for qRT-PCR. Red bars denotes "PCa patient" (n = 7) and grey bars denotes "BPH patient" (n = 7). Experiment performed on the same day for each patient, in triplicate.

References

1. Shirazi, H. S.; Chan, K. M.; Rouget, J.; Ostrikov, K.; McNicholas, K.; Li, J.; Butler, L.; Gleadle, J. M.; Vasilev, K.; MacGregor, M., Plasma enabled devices for the selective capture and photodynamic identification of prostate cancer cells. *Biointerphases* **2020,** *15* (3), 031002.

2. Chan, K. M.; Vasilev, K.; Shirazi, H. S.; McNicholas, K.; Li, J.; Gleadle, J.; Macgregor, M., Biosensor device for the photo-specific detection of immuno-captured bladder cancer cells using hexaminolevulinate: An ex-vivo study. *Photodiagnosis and Photodynamic Therapy* **2019**.
